# Participatory Action Research on the Impact of Community Gardening in the Context of the COVID-19 Pandemic: Investigating the Seeding Plan in Shanghai, China

**DOI:** 10.3390/ijerph18126243

**Published:** 2021-06-09

**Authors:** Huaiyun Kou, Sichu Zhang, Wenjia Li, Yuelai Liu

**Affiliations:** 1 College of Architecture and Urban Planning, Key Laboratory of Ecology and Energy-Saving Study of Dense Habitat of Ministry of Education, Tongji University, Shanghai 200092, China; khy@tongji.edu.cn; 2 Library and Information Centre, Shanghai Urban Construction Vocational College, Shanghai 200433, China; zhangsichu@succ.edu.cn; 3 College of Communication and Art Design, University of Shanghai for Science and Technology, Shanghai 200093, China; liwenjia@usst.edu.cn

**Keywords:** community gardening, PAR, COVID-19 pandemic, mental health, community building

## Abstract

This study aims to examine the impacts of community gardening on the daily life of residents and the management organisation of pandemic prevention in the context of the COVID-19 pandemic, a major public health scourge in 2020. The research team applied a participatory action research approach to work with residents to design and implement the Seeding Plan, a contactless community gardening program. The authors carried out a study to compare the everyday conditions reflecting residents’ mental health of the three subject groups during the pandemic: the participants of the Seeding Plan (Group A), the non-participants living in the same communities that had implemented the Seeding Plan (Group B), and the non-participants in other communities (Group C). According to the results, group A showed the best mental health among the three; Group B, positively influenced by seeding activities, was better than Group C. The interview results also confirmed that the community connections established through gardening activities have a significant impact on maintaining a positive social mentality under extraordinary circumstances. From this, the study concluded that gardening activities can improve people’s mental health, effectively resist negative impacts, and it is a convenient tool with spreading influence on the entire community, so as to support the collective response to public health emergencies in a bottom-up direction by the community.

## 1. Introduction

Exposure to natural environments contributes to the improvement of human health, which has been proven by a variety of theories and experiments since the 1980s, such as stress recovery theory and attention restoration theory [1,2,3,4,5]. Following that, therapeutic landscapes have been widely used as an adjuvant treatment in hospitals, nursing homes, and other professional medical institutions in the developed countries in Europe and America [6]. Most of those projects take the form of healing gardens targeting specific groups, such as people with certain diseases, the elderly, and children [7,8]. Some researchers have proposed that the concept of therapeutic landscape needs to be expanded from hospitals to public areas as well as from special groups to the general population [9].

Community gardening is shown to be the type of activity which enable ordinary people to get close to nature in their daily life and promote the health of individuals and the environments [10,11,12,13]. Community gardening has different forms and primary functions in different regions and at different times. For example, allotment gardens in Western Europe provided food security for landless city dwellers in the early 20th century and then gradually transitioned into recreational gardens around 1990 [14,15]; P-patch programs developed in Seattle in the United States of America since the late 1970s leased residents pieces of land to grow fruits, vegetables, and flowers, also as a mean to establish and promote neighbourhood connections [16,17]; the Urban Agricultural Garden in Japan took the form of both small farms in the urban built areas mainly for children and the elderly, and relatively large-scale farmlands in the inner and outer suburbs for farming experience activities and idyllic vacations [18]. The American Community Gardening Association (ACGA) gives a broad definition of the community garden as ‘any piece of land gardened by a group of people’, and ‘it can grow flowers, vegetables or community’ [19].

In China, the physical environment of most residential neighbourhoods in cities, including those central garden and green space scattering around the houses and buildings are managed by property management companies [20]. Resident-participatory gardening has emerged only since the 2010s [21]. Due to the high population density and the limited number of available lots, community gardens are mainly for horticultural display, health education, and the beautification of the residential environment [22]. In recent years, with the support of local governments and universities, community gardening has developed and spread rapidly as a way of environmental renewal for old neighbourhoods and a means of capacity building for communities [23,24].

The research team launched the Community Garden Initiative in China in 2014, aiming to create a green and healthy environment through collaborative horticultural activities by public participation. The initiative contains five stages: first, researchers took the lead in guiding residents’ involvement in gardening step by step; second, to engage enterprises and NGOs in community garden building with residents; third, to foster community leaders as pioneers to guide residents’ gardening; fourth, to encourage communities to carry out gardening activities independently, and; fifth, to develop the Initiatives to a community-oriented gardening program called the Seeding Plan. The ultimate goal of the initiative is to develop community gardening into a public campaign incorporating the whole society with the assistance of researchers. It is a progressive process with decreasing interventions from the outside and increasing autonomous governing within the community. It has integrated nearly 70 community gardens, going beyond the city of Shanghai to a number of cities across the country till 2019 when we conducted surveys on the effects of community gardens. The results confirmed a reinforcement on community cohesion and also demonstrated the greater influence of community gardening on mental health. The outcomes of the research have already been published [25].

The implementation of the Community Gardening Initiative was badly affected by the outbreak of coronavirus in early 2020. Most cities in China launched a first-level response to major health emergencies at the end of January 2020. Almost all the public places were shut down, including subways, buses, shopping malls, restaurants, bars, scenic spots, parks, museums, auditoriums, community centres, etc. The duration of the national holiday around the Spring Festival (24–31 January 2020) was extended to make sure people stay in place put for the prevention and control of the disease. Benefitting from gated and enclosed neighbourhoods design, the community became the frontline for epidemic prevention, with strict regulations on the entry and exit of neighbourhoods. Some neighbourhoods also imposed a period for daily shopping. Residents were encouraged to stay at home and cut down on outdoor activities. The peak weeks of the coronavirus outbreak vary from city to city, with about three months of strict social distancing in general. Even after the lockdown ended, people had to become accustomed to a new normal when living with the COVID virus and preventive measures of various degrees have become a part of daily life. The Seeding Plan, on the one hand, as the late stage of the entire Community Garden Initiative, was inevitably affected by the pandemic; but on the other hand, its contribution to the capacity building and mental health of the community was also highlighted. Therefore, the research and implementation of community gardening had to adapt to the situation for pandemic prevention.

The research team employs a participatory action research (PAR) framework, a type of applied social research aiming to pursue practical solutions to the situation; meanwhile, to advance scientific knowledge when the researcher and the researched acting together [26]. The PAR approach starts with the collective inquiry of what needs to be improved and then actions to solve the problem and change the status quo are implemented. PAR undergoes a process of a self-reflective spiral with planning, acting and observing, reflecting, replanning, etc. [27]. During the process, participants act as coresearchers in selecting the research topic, organising the action, and analysing the results, by which PAR blurs the line between the roles of researcher and participant and reconceptualises the research itself as a social practice [28,29]. After the outbreak of the COVID-19, community garden activities that had lasted for six years were suspended and waiting for a suitable way to resume. With the application of the PAR methods, the actual needs of participants in the context of the pandemic can be identified to make adjustments to the plan, when timely feedbacks can be obtained during the cooperative implementation. The action manual made by Peter Reason provides guidance and case references for the research in terms of the theoretical basis, research ethics, plan formulation, methodologies and skills of organisation and practice, etc. [30].

The research team restarted the Seeding Plan in mid-February 2020. This paper aims to investigate the impacts of community gardening on the daily life of residents and the organisation of community disease prevention in the context of the COVID-19 pandemic. Questionnaire surveys and comparative analyses were conducted in August 2020 to examine the daily situation of residents during the program. Detailed interviews were carried out to evaluate how the implementation of the Seeding Plan influenced the organisation of the community prevention of the disease. The research results show that gardening activities are a good way that enables access to nature and social interactions that can soothe people’s psychological stress during a major public health emergency. With easy implementation and dissemination through the community network, gardening contributes to improved mental health for the community to respond to health emergencies in a bottom-up process.

## 2. Materials and Methods

PAR is not a method at the micro level but rather a guide to creating the circumstance of accumulating knowledge and tracking change [31,32]. Robin McTaggart provided nine key principles guiding participatory action research: identifying the individual and collective project; distributing power; changing the culture of working groups, institutions, and society; acting and reflecting; unifying the intellectual and practical project; producing knowledge; engaging the politics of research action; employing methodological resources; creating the theory of the work [33]. Following these principles, the research team carried out the Seeding Plan in four phases: first, in the early stages of the pandemic, the research team exchanged views with participants about their daily life and needs, exploring the possible role the Seeding could play within communities; second, given the requirements for disease control, the research team worked with participants to design, organise, and implement the Seeding Plan in a contactless way; third, after adapting the initiative, it had a nationwide rolled out; fourth, the research team evaluated the outcome and solicited feedback, then further refined and improved the methodology and the goals. 

### 2.1. Inquiry and Planning

The six years of experience in the Community Garden Initiative laid a solid research and personnel foundation for the Seeding Plan, which had already started before the pandemic outbreak. In the initial stage of Shanghai’s lockdown, a research team comprising a core group of 11 researchers and 30 gardening activists from the communities held online meetings to identify the problems and needs of the residents, then laid out the objectives and action plans. Pertinent problems became relevant in the following two features: the depressive moods accumulated among the residents directly caused by the lockdown had no proper way to be released; social distancing led to distrust among neighbours. Therefore, the team focused on the two topics of ‘how to resume gardening activities during lockdown’ and ‘how to restore human interactions through gardening’. People being isolated for such a long time acquired a cave mentality, tending to take a long time to sociability. The idea of a contactless seeding action then came out pursuing ‘building of trust and seeding of hope’. The plan was set to restore communication between neighbourhoods through a non-contact form of seed sharing and planting and bring vitality into daily life.

The research team designed various contactless ways to distribute seeds and planting schemes, thus establishing an implementation plan involving collaborations among the researchers and participants, with the engagement of the active members from the neighbourhoods. Within two weeks, more than 400 participants joined the online social networking of the Seeding Plan for requesting technical support.

### 2.2. Organisation and Action

In the beginning phase, the team encouraged residents who already had seeds or those with access to seeds to start planting on their balconies, hallways, or vacant lots around their houses. The research team provided a few tips on gardening indoors, plant conservation, and other information through online guidance and Q&A sessions. At the same time, the team launched the Seed Moving Ark action to distribute seeds and provide year-round planting guidance to the participants who had already mobilised at least 10 households to join the action.

**Figure 1:**
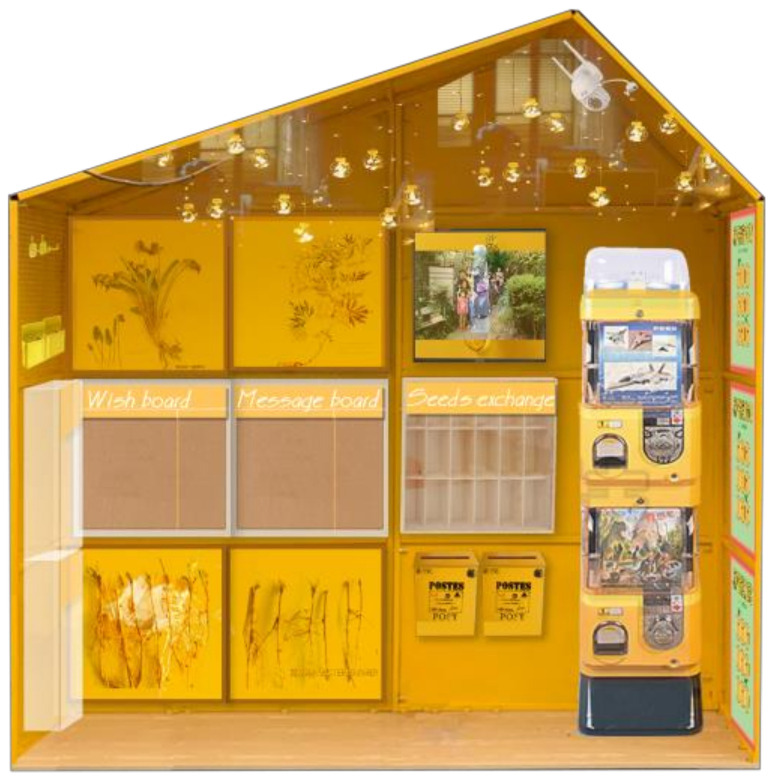
The design of the seed relay station.

The team designed a device called ‘seed relay station’, a simple wooden shelf to place seeds, envelopes and post-it notes, etc. Participants shared seeds from the shelf in a contactless way during the epidemic and thus established a new way to social interact (Figure 1). The first seed relay station was placed in the Knowledge and Innovation Community Garden, with 50 seed bags of vegetables available for residents and people working nearby. The recipients had to leave their personal information to receive free seeds. Several participants set up simple versions of seed relay stations in their neighbourhoods. They even tested several plans and then constructed the stations to reduce the cost of the station to enhance their attractiveness to potential participants as well as to improve the technical viability to leave messages and information. Finally, the establishment of about 50 stations in different neighbourhoods sustained the seeding relay and its expansion to a greater number of residents and communities.

### 2.3. Adjustment and Dissemination

The Seeding Plan also established a loop of feedback, adjustment, and promotion. Online salons met constantly to exchange planting experience and discuss technical issues. For example, the construction of the first batch of relay stations from paper boards for cost reduction meant they could only be placed indoors. Through discussion, upgrading the stations for long-term and outdoor use proved more effective. The research team also organised other online activities, such as a ‘cloud reading club’ to enhance social cohesion among the community residents.

**Figure 2:**
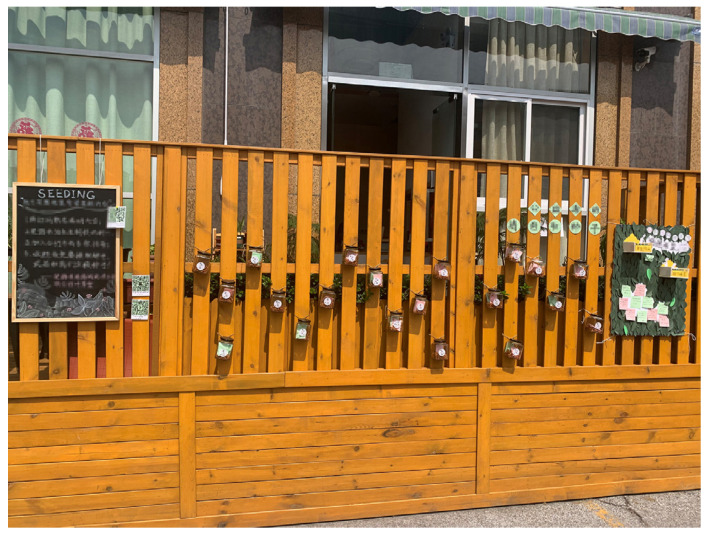
The seed relay station in front of Mr. Zhu’s Club. (The Chinese guidance of the Seeding activity in the photo is pixelated according to the publication requirement.)

As experience accumulated, the participants gradually grew into independent organisers and contributed to the expansion of their influence. Ms. Zhang, a retiree and member of the team, set up a seed relay station in her courtyard, organised garden tours and a neighbourhood market. Several households in her community have joined her. Mr. Zhu, a club owner, organised his employees to start planting activities and set up a seed relay station in front of his shop while also mobilising other shops along his street to participate in the Seeding Plan (Figure 2).

Seeding incorporates the meaning of ‘growing’, which indicates the nurturing of positive actors to spread the idea of actions and expanding the ranks of participants. In this process, the Seeding Plan had received support from horticultural, woodworking, landscape design, and short-video companies, official and folk media, community building groups, etc. They offered seeds, seedlings, timbers, video recording, and publicising ideas. Through six months of operation, the league of Seeding Plan had been formed with its branches in a dozen provinces and municipalities including Beijing, Tianjin, Guangdong, etc.

### 2.4. Evaluation and Feedback

After running for six months and undergoing an expansion nationwide, the research team conducted surveys to see how the Seeding Plan affected people during the outbreak of coronavirus.

The questionnaire survey employed means to obtain the data of the residents’ daily status during the lockdown. The influence of seeding activities can be learned by a comparison of the distinction between the Seeding Plan’s participants and non-participants. The team designed a questionnaire that asked eight questions to cover the changes to three aspects of individual status, social status, and environmental conditions (Table 1 and Appendix A). A scale from −4 to 4 reflected the degree of change, with 0 as the median value indicating no change, −4 indicating a significant negative change, and 4 indicating a significant positive change. Taking into account that the participants in seeding activities may share some common personality traits such as being more positive and enthusiastic, as well as the lockdowns impacts on people’s attitudes towards life, the questionnaire included a question to measure the self-perception of residents’ degree of optimism before and after the epidemic (Appendix A). To get more feedback about the Seeding Plan, an open question was also put at the end of the questionnaire to Group A, asking for comments and suggestions on the Seeding Plan (Appendix A). 

The research team distributed online questionnaires to three respondent groups: those who participated in the Seeding Plan (Group A), those who do not participate but live in the same communities where the Seeding Plan is implemented (Group B), and those who live in other communities without implementing the Seeding Plan (Group C). Investigating participants and non-participants in the same neighbourhoods that have implemented the Seeding Plan could avoid the bias caused by the difference in region and neighbourhoods, while other neighbourhoods which do not participate in the plan could be perceived as randomised control group without being influenced by the Seeding Plan’s activities. 

Questionnaires to group A were distributed in the Seeding Plan group in WeChat (a social media prevalent in China). Participants were encouraged to spread the questionnaires to those participants who were not in the WeChat group. The research team also asked participants to forward the questionnaires to their neighbours who did not participate in seeding activities (Group B). Questionnaires to group C were distributed online to the public via Questionnaire Star, which is a professional online questionnaire platform for surveying, evaluation, and voting (https://www.wjx.cn/, accessed on 8 June 2021). The questionnaire was distributed online and could not be submitted unless it was completed. Consequently, 10 days after the questionnaires were sent out, 1154 valid responses were collected, including 552 from Group A, 396 from Group B, and 206 from Group C. More than 30 communities that have implemented the Seeding Plan across the country participated in the survey. Compared with the whole amount of about 5000 participants and 300 communities that implemented the Seeding Plan, around 10% of the participants were involved in the questionnaire survey. As the questionnaire was distributed online through the sharing among social media groups, it is impossible to calculate the total number of questionnaires issued. The concept of response rate does not apply to this survey context.

**Figure 3:**
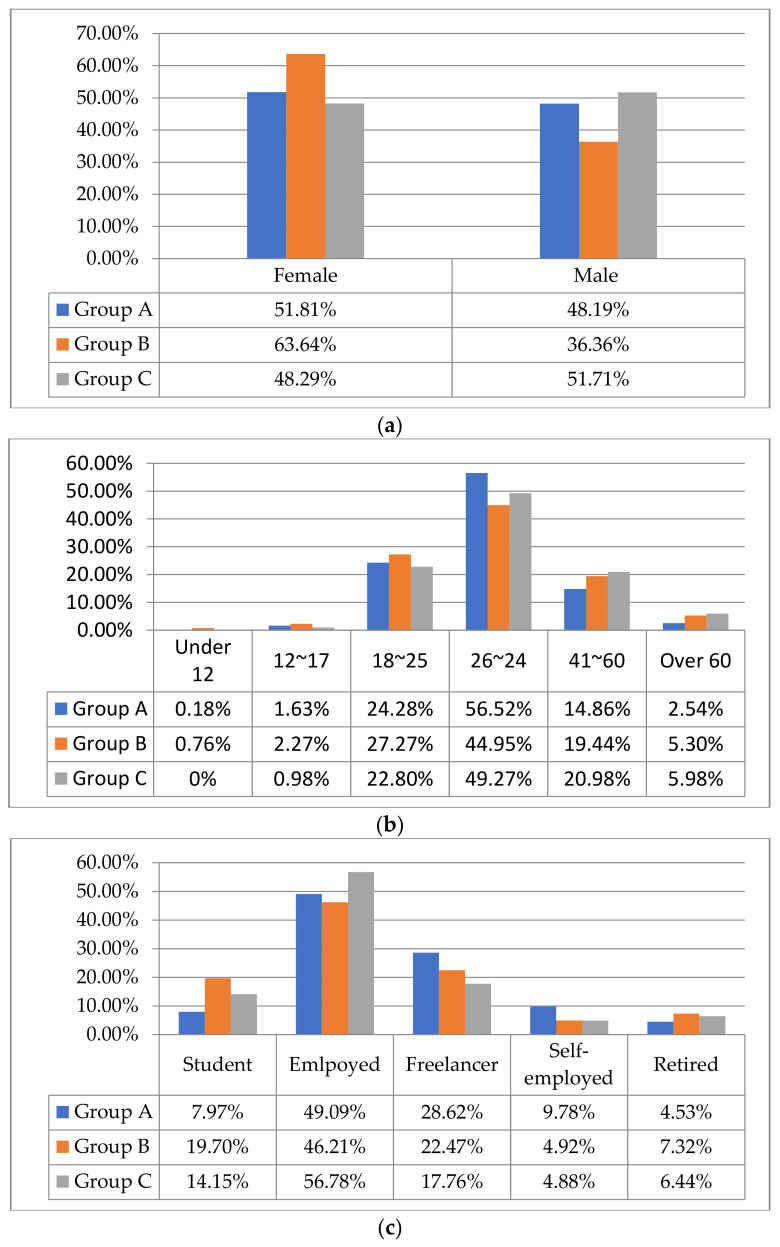
(**a**) Gender comparison; (**b**) age comparison; (**c**) occupation comparison.

The basic information of the samples in terms of gender, age, and occupation shows an equal distribution among the three groups, which makes the data comparable (Figure 3a–c). Comparing the data from the same period in 2019, the proportion of the employed who participated in community garden activities has increased significantly, from 54% to 87.5%, and the proportion of retirees has decreased from 39% to 4.5%, reflecting that the lockdown during the pandemic for the first time propelled the engagement of the working population in community activities [25].

The conducting of semi-structured interviews furthered the understanding of the impact of seeding activities on the overall organisation to halt the spread of the coronavirus for the whole neighbourhoods (Appendix B). 18 people were interviewed, including active members of community seeding actions, general participants, and social workers in the community. Given that the Seeding Plan is an initiative involving individual participants, neighbourhood committees (lowest level of governance in China) may not be aware of the existence of such activities. Therefore, the interviewees are not necessarily from neighbourhood committees, but those who are familiar with the situation of the whole community.

## 3. Results and Discussion

### 3.1. The Means Based on Statistics of Questionnaires

The means of the three groups on the changes in residents’ daily lives and degree of optimism during the lockdown period are as follows (Table 2 and Table 3).

The research team used SPSS to analyse the dispersion degree of the mean values of the results of the three groups’ questionnaires (Table 4). 

The standard deviations of the three groups were very close and with no obvious deviation, reflecting low dispersion, which meant that the probability of decisive difference among each group of samples was comparably low, and the statistic outcomes were reliable.

Correlation analysis was carried out with *p*-values. The *p*-values of each item were all less than 0.01 (Table 4), which confirmed the rejection of the null hypothesis; it proved that at the 1% probability level, the answers of the three groups of respondents on each question were different.

### 3.2. Comparison of the General Status of Daily Life during the Lockdown

Comparing the means of Group A and Group B in Table 2, we can conclude that based on either the total mean (2.31 vs. 1.17) or the mean of the eight indicators, values of the Seeding Plan participants showed significantly higher than that of non-participants in the same neighbourhoods. This result showed that seeding activities had significant positive effects on the participants’ physical and mental health, social interactions, living and working environments during the outbreak period of the virus. Further examination of the means of optimism level shows that the Seeding Plan participants were more optimistic both before and after the outbreak than non-participants (Table 3), meaning that the personalities of the group remained relatively positive, which is one of the reasons why the mean difference in daily quality of life between participants and non-participants was so distinct.

Comparing the means of Group B and Group C, values of non-participants in Seeding neighbourhoods were significantly higher than those in non-Seeding neighbourhoods (Table 2), reflecting the positive impact of seeding activities on the entire community.

The mean values of both the participants and the non-participants were statistically higher than the median value of zero (Table 2), reflecting the overall positive quality of daily lives during the outbreak. This result did not seem to be consistent with the public knowledge about the pandemic, but it did reflect the objective situation in China during the lockdown in 2020: except for Hubei Province, the ‘epicentre’ of the epidemic, the infection rate in other regions was extremely low. The strict prevention and control measures such as shutting down companies, factories, and schools and instituting remote working, enabled a slowdown of the pace of both living and working for the whole society. The time for leisure and self-entertainment increased and these activities were organised around neighbourhoods. With more time to enjoy individual and family life, the physical and mental health and social interactions, as well as the environmental conditions, all improved. It should be noted that because the Seeding Plan was first initiated in Shanghai, the major participants were from Shanghai (72%), and in communities in other major cities across the country, where the living conditions were relatively better off than the national average. Therefore, the statistic values are not expected to represent the general situation in the entire country. However, the comparison of differences between participants and non-participants in the same communities excluded the influence of regional and community differences and was effective in measuring the results of the Seeding Plan.

### 3.3. Comparison of the Individual Indicators during the Lockdown

**Figure 4:**
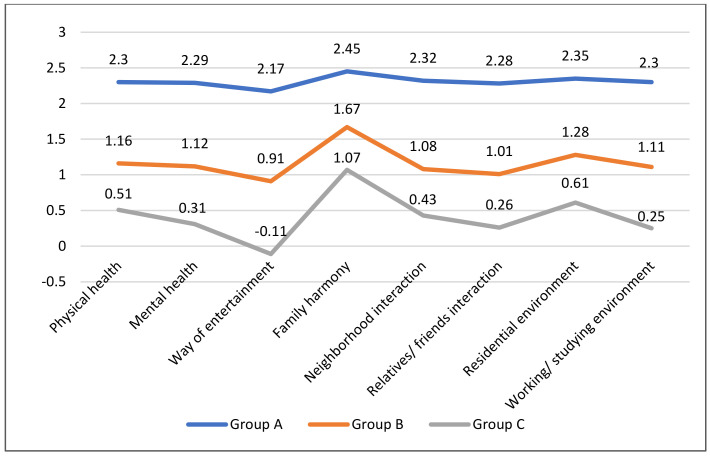
Comparison of the means of indicators.

The comparison of values of each indicator showed a rather consistent trend for both participant group and non-participant groups: the most significant change existed in family harmony (2.45/1.67/1.07), followed by residential environment (2.35/1.28/0.61), with a minimum change in the improvement of entertainment (2.17/0.91/−0.11); while the statistics of other indicators were relatively close. It can be inferred that social distancing contributed to the improvement of family relationships and the residential environment. Although leisure time increased, the forms of leisure and entertainment were limited by the travel restrictions and social distancing, and consequently, it was the only negative factor in this questionnaire (Figure 4). 

The difference between group A and group B reflected the extent to which the seeding activity influenced the participants’ rating of their daily life. Many comparative assessments did not change that much according to the difference in Table 2: the impact on the family/friend interactions (1.27), the form of entertainments (1.26), and the neighbourhood interactions (1.24) were slightly higher; the influence on physical health (1.14), mental health (1.17) and working and study environments (1.19) were in the middle; while that on family harmony (0.78), family, and residential environments (1.07) were relatively weak. In general, the influence of seeding on family harmony was relatively smaller than others. This is easy to understand given the activity is essentially focusing on the restoration of community interactions The results also reflected the contactless approach of seeding activities, which, though not as effective as offline community gardening to the environmental improvement, did provide outward recreational opportunities to promote social interactions during the lockdown period. Therefore, seeding is a form of social entertainment that is not limited by space or distance which helps maintain physical and mental health during the lockdown. The answers to the open-ended question of the questionnaire further confirm this positive role. Provided here is an answer from Ms. Wei:

‘Seeding helped many of our families to reconnect and return to nature by farming during the pandemic, unease the stress from the outbreak and bring us new understanding and hope of life. Staying at home for a few months, every day I share with other seeding participants in the WeChat group the process of the seed growth, and I sensed sincerity and kindness flowing among people. I kept thinking about the seed relay station all the time and can’t wait to go there to share with others as soon as I obtained new seeds. I have started to talk to my neighbours that I didn’t know about before. Now every time we meet, I would say hello to them.’

The indicator difference between Group B and Group C was about the same. This comparably slight difference in the family indicator was ascribed to the interactive nature of the activity with which people with extroverted personalities tend to be more comfortable. A comparably larger difference could be observed in the entertainment indicator. A possible explanation was that although Group B did not participate directly in seeding activities, the lack of entertainment during this isolation period increased people’s mental sensitivity, thus the perception of the activity seemed more obvious. One of the members of group B wrote:

‘Seeding is a meaningful thing, I was meant to join in your activities but I couldn’t because of my job. I feel lucky to be aware of the existence of such kind of public activities and are moved by the efforts and dedication people have invested in it. I hope everyone will keep their dreams, and make the Seeding Plan better!’

### 3.4. Analysis of the Community Organisations during the Lockdown

The interviews with active participants, general participants, and social workers in the communities have generally confirmed the positive impact of seeding activities on the COVID-19 prevention measures and other community activities. Regarding the impact on pandemic prevention work, 15 out of the 18 respondents believed that seeding activities provided exchange opportunities for people in the same neighbourhoods to understand each other better, which, in turn, facilitated the management organisation of pandemic prevention work within the community. They gave examples of participants spontaneously donating masks and disinfectants to the community and sharing other pandemic prevention reserves. Two other respondents deemed there were neither positive nor negative impacts of seeding activities. However, the other respondent held a negative view towards the seeding activity, accusing it of producing more garbage. Regarding the impact on other activities in the community, 14 respondents believed that due to the foundation of seeding activities, more residents joined the online or offline activities in the neighbourhoods, therefore making it easier to organise other community activities. They also listed a few other activities such as cloud picnics, float production, parent–child activities, garbage sorting, and other activities. The other four respondents said they were not aware of other activities. 

It can be inferred that seeding activities can eliminate the anxiety of home isolation, increase trust between people, and help individuals to be more engaged in public affairs. Therefore, it has accumulated experience for community responding to major public emergencies in the future. When people use their balconies, doorways plus other private and semi-private spaces for sharing activities, the space boundary between private and public areas breaks down, which activates individuals’ concern about public affairs, thus promoting the establishment of community identity.

## 4. Conclusions

There are three characteristics of community gardening that make it prominent in public health emergencies: (1) community gardening and its easy access as a ubiquitous natural element in daily life match people’s increasing needs and longing for nature as a means to release pressures; (2) gardening as both a group and individual activity effectively adapts to spatial limitations imposed by lockdown and social distancing; (3) the technical issues involved in gardening provides a good medium for communication between neighbourhoods.

The study confirmed the positive role of community gardening activities to promote mental health and provided a viable strategy for the community to respond properly to public health emergencies. This is demonstrated in three aspects:

First, gardening activities have direct or indirect impacts on people’s mental health, which becomes particularly relevant in the context of public health emergencies. The data on the changes in the quality of daily life, whether in personal, social, or environmental dimensions, were collected by the descriptions of subjective feelings, not by quantitative measurements of objective factors. Therefore, they were results from the subjective perception of the respondents, which belongs to mental status.

Second, neighbourhood connections established through gardening activities benefit the organisation of virus spread prevention measures within the community, and its bottom-up way helps the community to respond properly to public health emergencies. With the least restrictions on space and distance, the Seeding Plan can quickly spread among individuals to the whole community, as reflected by the contrast between Group B and Group C. The interview surveys also confirmed its flexibility in organisation and implementation forms and its adaptability to the ways of human interactions under the lockdown period, which improved residents’ sense of belonging and the capacity building of the community. 

Third, organisation and participation in horticultural activities is an effective way to relieve negative emotions in the context of public health emergencies since gardening is an activity that enables close contact with nature to release pressure, as well as having flexible application in space and acting as a convenient medium of communication. The comparison of data from the questionnaire and interview surveys confirmed this conclusion. 

The limitation of the study exists in two aspects: (1) questionnaires of group C were randomly distributed online; therefore, they did not evenly cover the populations of all provinces and cities in China; the respondents with the characteristics of groups A and B could not be excluded from group C, but from the *p*-value analysis of the samples, we can infer no population with characteristics of group A and B; (2) the social and interactive nature of the Seeding Plan means the activity tends to attract the participation of those who innately have a more positive and optimistic personality, leading to a possible biased high baseline value of the survey data in seeding communities. However, it does not affect the general conduction of comparative research. Instead, it supports the idea of community gardening as a public health strategy, as it can rally optimistic people to develop a positive driving force to promote personal, familial, and community health as well as the overall neighbourhoods’ environment. This also matches the ultimate goal of the Seeding Plan: to make gardening activities a collective action initiate from bottom to up by the community and to have fun.

## Figures and Tables

**Table 1:** Questionnaire design.

No.	Indicators		Scoring	Respondents
1	Changes in personal circumstance	Physical health	−4 to 4	Group A: participants Group B: non-participants in Seeding communities Group C: non-participants in non-seeding communities
2		Mental health		
3		Way of entertainment		
4	Changes in social situation	Family harmony		
5		Neighbourhood interaction		
6		Relatives/friends interaction		
7	Changes in environmental conditions	Residential environment		
8		Working/studying environment		
9	Degree of optimism	Before epidemic		
10		After epidemic		

**Table 2:** Mean values of changes in the quality of daily life.

No.	Indicators		Group A (Participants)	Group B (Non-Participants in Seeding Communities)	Group C (Non-Participants in Non-Seeding Communities)	Difference between A and B	Difference between B and C
	Number of respondents		552	396	206	-	-
1	Changes in personal situation	Physical health	2.3	1.16	0.51	1.14	0.65
2		Mental health	2.29	1.12	0.31	1.17	0.81
3		Way of entertainment	2.17	0.91	−0.11	1.26	1.02
4	Changes in social situation	Family harmony	2.45	1.67	1.07	0.78	0.60
5		Neighbourhood interaction	2.32	1.08	0.43	1.24	0.65
6		Relatives/friends interaction	2.28	1.01	0.26	1.27	0.75
7	Changes in environmental conditions	Residential environment	2.35	1.28	0.61	1.07	0.67
8		Working/studying environment	2.3	1.11	0.25	1.19	0.86
Total			2.31	1.17	0.42	1.14	0.75

**Table 3:** Mean values of optimism.

No.	Items	Group A (Participants)	Group B (Non-Participants in Seeding Communities)	Group C (Non-Participants in Non-Seeding Communities)
	Number of respondents	552	396	206
9	Before epidemic	2.6	1.77	1.49
10	After epidemic	2.63	1.85	1.4

**Table 4:** Analysis of variance.

	Group (Mean ± Standard Deviation)			*F*	*p*
	Group A (*n* = 552)	Group B (*n* = 396)	Group C (*n* = 206)		
1	2.30 ± 1.79	1.16 ± 1.87	0.51 ± 1.70	85.937	<0.01
2	2.29 ± 1.78	1.12 ± 1.91	0.31 ± 1.97	100.113	<0.01
3	2.17 ± 1.93	0.91 ± 2.04	−0.11 ± 1.98	113.843	<0.01
4	2.45 ± 1.67	1.67 ± 1.77	1.07 ± 1.70	56.414	<0.01
5	2.32 ± 1.73	1.08 ± 1.86	0.43 ± 1.64	109.231	<0.01
6	2.28 ± 1.80	1.01 ± 2.10	0.26 ± 1.80	106.367	<0.01
7	2.35 ± 1.76	1.28 ± 1.84	0.61 ± 1.63	87.516	<0.01
8	2.30 ± 1.83	1.11 ± 1.98	0.25 ± 1.74	105.662	<0.01
9	2.60 ± 1.65	1.77 ± 1.87	1.49 ± 1.80	42.092	<0.01
10	2.63 ± 1.58	1.85 ± 1.93	1.40 ± 2.00	44.109	<0.01

## Appendix A. Questionnaire for Residents’ Daily Status during the Lockdown

1. Basic information
  (1) Gender: [A] Male [B] Female
  (2) Age: [A] Under 12 [B] 12–17 [C] 18–25 [D] 26–40 [E] 41–60 [F] Over 61
  (3) Occupation status: [A] Student [B] Employed [C] Freelancer [D] Self-employed [E] Retired
2. Changes in the quality of daily life during the lockdown

How do you feel about the changes of the following situations during the lockdown? Please rate them according to the extent from −4 to 4: ‘0′ (no change), ‘4′ (apparently positive change), ‘−4′ (apparently negative change).

**Question**		**Score**								
		**−4**	**−3**	**−2**	**−1**	**0**	**1**	**2**	**3**	**4**
1	Your physical health	□	□	□	□	□	□	□	□	□
2	Your mental health	□	□	□	□	□	□	□	□	□
3	Your way of entertainment	□	□	□	□	□	□	□	□	□
4	Your family harmony	□	□	□	□	□	□	□	□	□
5	Your neighbourhood interaction	□	□	□	□	□	□	□	□	□
6	Your interaction with relatives and friends	□	□	□	□	□	□	□	□	□
7	Your residential environment	□	□	□	□	□	□	□	□	□
8	Your working or studying environment	□	□	□	□	□	□	□	□	□

3. People’s self-awareness of attitudes towards life before and after the Pandemic

How do you feel about your optimism about life before and after the pandemic? Please rate them according to the extent from −4 to 4: ‘0′ (no change), ‘4′ (apparently positive change), ‘−4′ (apparently negative change).

**Question**		**Score**								
		**−4**	**−3**	**−2**	**−1**	**0**	**1**	**2**	**3**	**4**
9	Your optimism about life before the pandemic	□	□	□	□	□	□	□	□	□
10	Your optimism about life after the pandemic	□	□	□	□	□	□	□	□	□

4. What do you think is the biggest gain for you from participating in seeding activities? Do you have any comments and suggestions on the SeedingPlan? (for Seeding Plan participants) ___

## Appendix B. Interview for Community Organisations during Lockdown

*Basic Information*

1. When did the Seeding Plan start in your neighbourhood?
  □ Before the COVID-19 pandemic; □ during the COVID-19 pandemic
2. Number of people in your neighbourhood who participated in Seeding Project ___
3. Number of people living in your neighbourhood ___

*Community organisations during lockdown*

1. Is there any impact of the Seeding Plan on the pandemic prevention work of your neighbourhood? May you give me an example?
2. Is there any impact of the Seeding Project on the carry out of other activities in your neighbourhood? May you give me an example?

## References

[1] Ulrich R. (1984). View through a window may influence recovery from surgery. Science.

[2] Ulrich R.S. (2002). Health benefits of gardens in hospitals. Proceedings of Paper for Conference.

[3] Kaplan S. (1995). The restorative benefits of nature: Toward an integrative framework. J. Environ. Psychol..

[4] Kaplan R., Kaplan S. (1989). The Experience of Nature: A Psychological Perspective.

[5] Velarde M.D., Fry G., Tveit M. (2007). Health effects of viewing landscapes–Landscape types in environmental psychology. Urban For. Urban Green..

[6] Marcus C.C., Barnes M. (1999). Healing Gardens: Therapeutic Benefits and Design Recommendations.

[7] Moore R., Cosco N. (2005). Well-being by nature: Therapeutic gardens for children. Proceedings of Latis Forum on Therapeutic Gardens.

[8] Cimprich B. (1992). Attentional fatigue following breast cancer surgery. Res. Nurs. Health.

[9] Mooney P.F., Chen J. (2009). The International Development of Therapeutic Landscapes. Chin. Landsc. Archit..

[10] Soga M., Cox D.T.C., Yamaura Y., Gaston K.J., Kurisu K., Hanaki K. (2017). Health Benefits of Urban Allotment Gardening: Improved Physical and Psychological Well-Being and Social Integration. Int. J. Environ. Res. Public Health.

[11] Clarke L.W., Jenerette G.D. (2015). Biodiversity and direct ecosystem service regulation in the community gardens of Los Angeles, CA. Landsc. Ecol..

[12] Hou J., Johnson J.M., Lawson L. (2009). Greening Cities, Growing Communities.

[13] Alaimo K., Beavers A.W., Crawford C., Snyder E.H., Litt J.S. (2016). Amplifying health through community gardens: A framework for advancing multicomponent, behaviorally based neighborhood interventions. Curr. Environ. Health Rep..

[14] Poole S. (2006). The Allotment Chronicles: A Social History of Allotment Gardening.

[15] Qian J. (2011). The Comparison of Allotment Garden in Europe and Community Garden in the United States. Mod. Urban Res..

[16] Diers J. (2004). Neighbor Power: Building Community the Seattle Way.

[17] Hou J., Grohmann D. (2018). Integrating community gardens into urban parks: Lessons in planning, design and partnership from Seattle. Urban For. Urban Green..

[18] Higashi M., Li J. (2018). Japanese Urban Agricultural Garden. Dev. Small Cities Towns.

[19] ACGA What Is a Community Garden?. https://web.archive.org/web/20071204082111/http://www.communitygarden.org/learn/.

[20] Zou H., Yu H. (2017). Urban Regeneration: From Space Production to Community Building—Taking Shanghai KIC Garden as an Example. Expand. Horiz..

[21] Qian J. (2011). The Absence of the Landscape: Community Gardens in the Context of Urban PIanning.

[22] Liu Y., Kou H. (2019). Study on the Strategy of Micro-renewal and Micro-governance by Public Participatory of Shanghai Community Garden. Chin. Landsc. Archit..

[23] Yu H. (2017). Back to the Life World and Lived World: A Case Study of Knowledge & Innovation Community Garden in Shanghai. Urban Rural Plan..

[24] Ma H., Ying K. (2016). Micro-rengeneration of community public: Exploring Approaches to Community Building in the Context of Organic Urban Regeneration in Shanghai. Time+ Archit..

[25] Kou H., Zhang S., Liu Y. (2019). Community-engaged research for the promotion of healthy urban environments: A case study of community garden initiative in Shanghai, China. Int. J. Environ. Res. Public Health.

[26] Whyte W.F. (1989). Advancing scientific knowledge through participatory action research. Sociological Forum.

[27] Kemmis S., McTaggart R. (2005). Participatory Action Research: Communicative Action and the Public Sphere.

[28] Baum F., MacDougall C., Smith D. (2006). Participatory action research. J. Epidemiol. Community Health.

[29] Salmen L.F. (1987). Listen to the People: Participant-Observer Evaluation of Development Projects.

[30] Reason P., Bradbury H. (2001). Handbook of Action Research: Participative Inquiry and Practice.

[31] McTaggart R. (1997). Guiding principles for participatory action research. Participatory Action Research: International Contexts and Consequences.

[32] Kidd S.A., Kral M.J. (2005). Practicing participatory action research. J. Couns. Psychol..

[33] McTaggart R. (1991). Principles for participatory action research. Adult Educ. Q..

